# Concordance between patient-centered and adaptive behavior outcome measures after applied behavior analysis for autism

**DOI:** 10.1186/s12887-022-03383-2

**Published:** 2022-05-27

**Authors:** Kristen R. Choi, Amin D. Lotfizadah, Bhumi Bhakta, Paula Pompa-Craven, Karen J. Coleman

**Affiliations:** 1 UCLA School of Nursing, 700 Tiverton Ave, Los Angeles, CA 90049 USA; 2 Department of Health Policy and Management, UCLA Fielding School of Public Health, 650 Charles E Young Dr S, Los Angeles, CA 90095 USA; 3 Department of Research & Evaluation, Kaiser Permanente Southern California, 100 S. Los Robles, Pasadena, CA 91101 USA; 4 Easterseals Southern California, 1063 McGaw Avenue, Suite 100, Irvine, CA 92614 USA

**Keywords:** Autism spectrum disorder, Applied behavior analysis, Patient-centered outcomes, Concordance, Measurement

## Abstract

**Background:**

Applied behavior analysis (ABA) is an evidence-based approach to autism spectrum disorder that has been shown in clinical trials to improve child functional status. There is substantial focus in ABA on setting and tracking individualized goals that are patient-centered, but limited research on how to measure progress on such patient-centered outcomes.

**Purpose:**

The purpose of this investigation was to assess concordance between patient-centered and standard outcome measures of treatment progress in a real-world clinical sample of children receiving ABA for autism spectrum disorder.

**Methods:**

This observational study used a clinical sample of children ages 3 to 16 years (*N* = 154) who received 24 months of ABA from an integrated health system. Concordance between three outcome measures after ABA was assessed using a correlation matrix: (1) patient-centered measures of progress on individualized treatment goals, (2) caregiver-centered measure of progress on treatment participation goals, and (3) the Vineland Adaptive Behavior Scales adaptive behavior composite.

**Results:**

There was limited concordance among measures at both 12 and 24 months of ABA. None of the patient-centered measures showed significant positive correlation with adaptive behavior composite difference scores at either 12 or 24 months, nor did the caregiver measure. The percentage of children achieving clinically meaningful gain on patient-centered goal measures increased between 12 and 24 months of ABA, while the percentage of children achieving clinically meaningful gains in adaptive behavior declined during the same time period.

**Conclusions:**

In a health system implementation of ABA, there was limited concordance between patient-centered and standard measures of clinically meaningful treatment progress for children with ASD. Clinicians should have ongoing dialogue with patients and parents/caregivers to ensure that interventions for ASD are resulting in progress towards outcomes that are meaningful to patients and families.

## Introduction

Autism Spectrum Disorder (ASD) is a developmental disorder that affects an estimated one in every 54 children [1]. ASD is characterized by difficulties with social interaction and communication and restricted or repetitive behaviors that interfere with activities of daily living [2]. Evidence-based treatment for ASD commonly includes applied behavior analysis (ABA), an intervention that uses a range of structured and naturalistic approaches grounded in basic learning principles and designed to teach functional skills [3–5]. Studies of ABA suggest that it can improve behavioral function for individuals with ASD, particularly when ABA is delivered intensively in early childhood [5]. Experts also believe that parent/caregiver involvement in ABA (e.g., communication with providers; reinforcing behavior goals outside of formal sessions; parent self-education) is necessary for improving child functional outcomes [6, 7]. A defining feature of ABA is its reliance on continual monitoring of progress towards treatment goals and periodic standardized assessments of improvement in symptoms and function. Although there are established guidelines for ABA and processes for measurement of treatment outcomes in controlled research studies [8], there is significant heterogeneity in actual delivery of ABA in real-world clinical care and how treatment outcomes are defined [9].

While there is a sizable literature on standardized ASD outcome measures (e.g., Vineland Adaptive Behavior Scales [10]), real-world measures of ABA treatment have received less study. There is substantial focus on setting individualized treatment goals in real-world ABA, but limited evidence about how to optimally measure progress on such individualized goals [11, 12] and what constitutes treatment “success” [13, 14]. Because there is a growing emphasis on patient-centered approaches to care and measurement of patient-centered outcomes in health service delivery, it is important to understand whether these real-world patient goal measures of progress agree with other validated measures of treatment outcomes.

Patient-centered outcomes are defined as treatment outcomes that are meaningful and important to patients, their caregivers, and their families, or striving to achieve “the best possible outcome based on each child’s personal characteristics and available supports” [15]. Tracking and improving patient-centered outcomes is a high priority in healthcare, and there is a growing focus in research on measuring patient-centered outcomes rather than only clinician- or healthcare-defined outcomes [16]. This movement to address patient-centered outcomes in research and practice is a needed change and has great potential to improve health services for individuals with autism. ABA emphasizes the importance of patient-centered outcomes when considering intervention goals [17]; however, evidence of success after ASD treatment has historically been defined as improved verbal, social, and intellectual abilities (e.g., IQ scores) and decrease or elimination of ASD symptoms rather than progress on patient-centered goals [15]. The number of individualized treatment goals that a child masters is widely used in practice as a patient-centered outcome measure, and these goals may be more likely to reflect treatment gains that are meaningful to patients and families.

Given the lack research on patient-centered outcomes after ABA, like skills for daily living or other individualized functional goals, there is a need to consider whether widely used individualized patient goal measures are concordant with traditional outcome measures used in ASD treatment research, such as adaptive behavior (e.g., Vineland Adaptive Behavior Scales), maladaptive behavior (e.g., Child Behavior Checklist), and cognitive abilities (e,g., Wechsler Intelligence Scale for Children®). Studies have suggested that ABA intervention hours have a positive association to both mastering a greater number of patient goals and behavior improvements on standard measures of adaptive or maladaptive behavior [4, 18–21]. However, there are no studies that have directly compared outcomes across standardized assessments and patient-centered outcomes for patients who have spent a sufficient length of time of ABA to expect outcomes to show clinically meaningful progress, generally considered to be at least 12 to 24 months of ABA [18]. The purpose of this study was to assess concordance between three outcome measures for children who received 24 months of ABA: (1) A patient-centered outcome measure of progress on individualized treatment goals, (2) a caregiver-centered outcome measure of treatment participation goals, and (3) the Vineland Adaptive Behavior Scales (Vineland-II) adaptive behavior composite (ABC), a standard measure of child adaptive behavior. These project aims arose from the theoretical framework for ABA which targets multiple domains of patient behavior and function and evidence suggesting that ABA improves multiple domains of outcomes simultaneously.

## Method

### Design and data

Data used for this investigation were part of a larger observational study designed to evaluate receipt of ABA by children with ASD receiving care in a large integrated health system [22]. Referrals for ABA originated within the health system, and ABA was then administered by a contracted external provider who returned reports about patient progress on goals every 6 months and ABA outcomes measured with the Vineland-II every 12 months to the patient’s electronic health record. The Vineland-II was required for insurance reimbursement. Patient demographic data from an ASD registry maintained by the health system were linked to patient ABA outcome data and service use characteristics from these records at baseline, 12 months, and 24 months of ABA treatment. The study was approved by appropriate Institutional Review Boards of investigators.

### Sample and setting

**Fig. 1:**
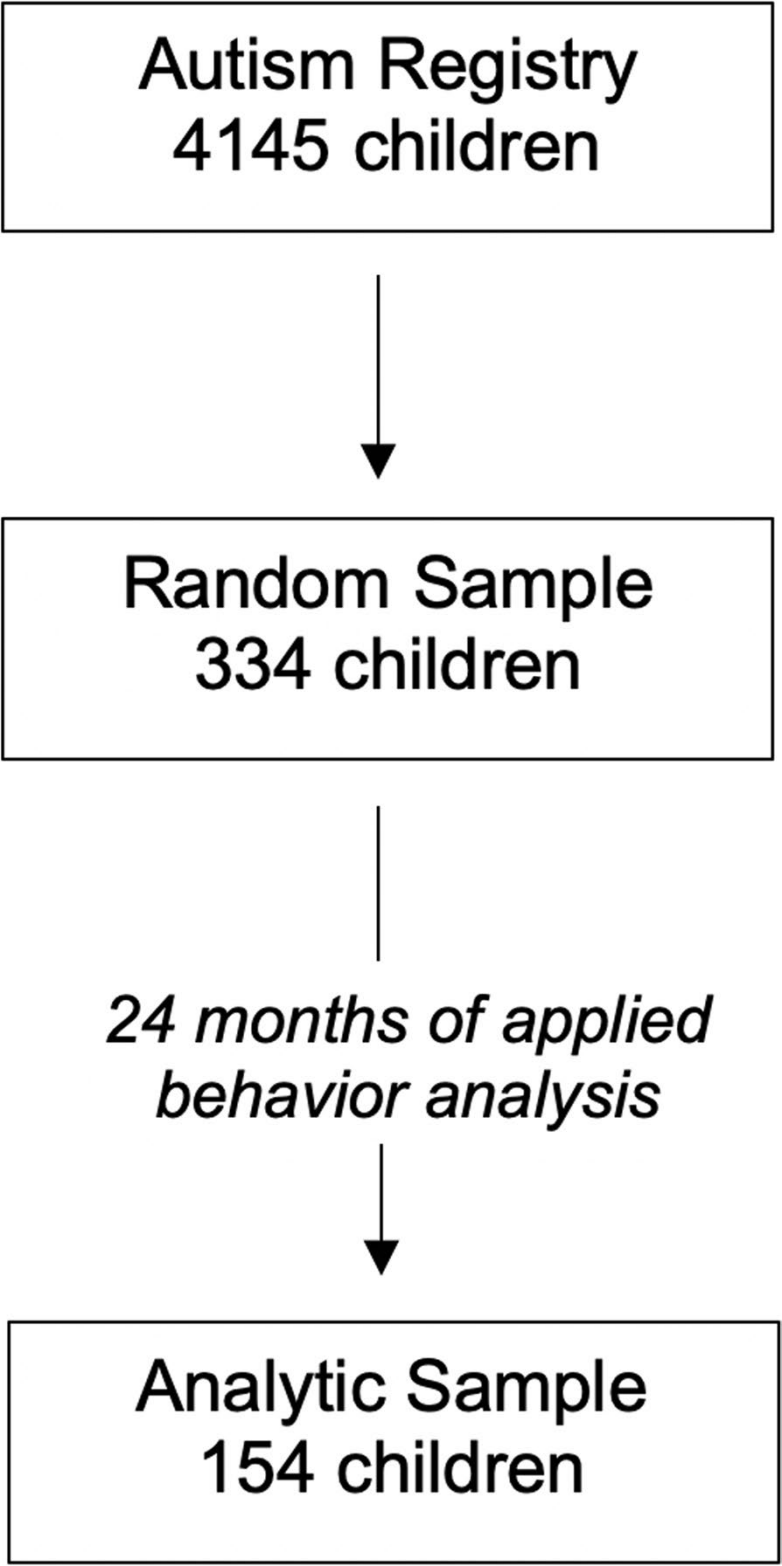
Sample Flow Diagram. Legend: This figure shows how the analytic sample was derived. There were 4145 children in the health system autism registry who received at least one referral for a new episode of applied behavior analysis (ABA) from 2016 to 2019. A random sample of 334 children was drawn from this sample for detailed electronic health record data extraction. Of this sample, 154 children received ABA for a full 24 months and met inclusion criteria for this analysis

The setting was an integrated health system in Southern California. The population of interest were 4145 children ages 3–17 years in an ASD registry who received a referral for a new episode of ABA between January 1, 2016 and October 31, 2019. From these 4145 children, 334 were randomly selected for electronic medical record review using a digital random sampling program with strata for age (3–6 years; 7–11 years; 12–17 years), gender (boys, girls), insurance type (Commercial, Medicaid), and race/ethnicity (White, Black, Hispanic, Asian, Other). Data were extracted from electronic health records for up to 24 months (or until the child discontinued ABA) following the patient’s baseline assessment. Because the purpose of the current investigation was to determine concordance between treatment outcome measures after a sufficient amount of time in ABA to observe clinically meaningful progress, but also to capture real-world patterns of treatment engagement among children referred for ABA, inclusion criteria were that the child received a full 24 months of ABA and had data on all three outcome measures. Consistent with practice guidelines for ABA and insurance reimbursement requirements for ABA in California [23], we assumed that children referred for ABA had a clinical indication for this service and thus included children of all ages. The analytic N was a subsample of 154 children who met these criteria (Fig. 1).

### Procedures

A protocol was developed by the co-authors to extract data for the following variables from electronic health record reports from the external ABA provider: past services received, hours or sessions of ABA prescribed per 6 months, hours or sessions of ABA received per 6 months, patient progress on individualized treatment goals, Vineland-II adaptive behavior composite, family characteristics not available in the autism registry (primary language spoken, parent marital status). These data were then linked to demographic registry data (gender, age, race/ethnicity, insurance type). Patient and caregiver progress on goals was reported every 6 months and adaptive behavior outcomes were reported every 12 months.

### Measures

This investigation focused on measures that were indicators of clinically meaningful improvement. There were three main outcomes that were compared for concordance: (1) patient progress on individualized treatment goals, (2) caregiver progress on education and participation in treatment goals, and (3) child adaptive behavior measured by the Vineland-II ABC 12-month difference scores. We examined dichotomous indicators of clinically meaningful progress for all outcome variables because dichotomous outcomes are usually preferred in making practice decisions [24].

#### Patient progress on goals

Patient progress on goals was measured using four domains of behavioral function used by the ABA provider in the health system under study: Expressive and receptive communication, pragmatic communication, behavior reduction/target, and self-help and daily living skills. These domains are those which ABA targets and where behavioral change would be expected over time in ABA.

For each of the four patient domains, Board Certified Behavior Analysts® (BCBAs®) and the child’s parent/caregiver set measurable, individualized goals for the patient at the time of baseline assessment. BCBAs® then documented patient progress on goals every 6 months in consultation with parents/caregivers (met, in progress, on hold, discontinued). Because the patients had different numbers of goals in various domains, we examined the number of goals met out of the number goals actively targeted (i.e., not on hold or discontinued) as a percentage for each goal domain and point in time. We examined whether or not patients had met at least 50% of their actively targeted goals in each domain and at each timepoint. Because there is no specific guidance on what constitutes clinically meaningful progress on patient goals, we selected 50% (half) as a threshold for this study.

#### Caregiver progress on education and participation goals

The ABA provider used by the health system also assessed caregiver education and participation in treatment as an individualized goal domain, assessed with the patient goals described above. We treated this goal measure separately from the four child goal measures because it targeted parents/caregiver behavior rather than child behavior. The caregiver education and participation goals were set and measured the same manner as the child goals described above. We again examined the number of goals met out of the number goals actively targeted (i.e., not on hold or discontinued) as a percentage for each goal domain and timepoint, plus a dichotomous indicator of clinically meaningful progress on caregiver education and participation (at least 50% of actively targeted goals met). We selected 50% as an indicator of progress because there are no existing standards for measures of progress in this domain.

#### Adaptive behavior

Child adaptive behavior was measured by the Adaptive Behavior Composite (ABC) on the Vineland Adaptive Behavior Scales-Second Edition (Vineland-II) [10, 25, 26]. Vineland-II responses were reported by parents or caregivers to the contracted behavioral interventionist, who then returned the scores within annual progress reports. This measure was selected because as an insurance reimbursement requirement, it was the only consistently measured, validated indicator of patient outcomes available in reports. The Vineland-II measures adaptive behavior that allow function in everyday life for individuals with developmental disabilities. The ABC is an overall composite measure of adaptive behavior based on Vineland-II subscales for communication, daily living skills, and socialization. An age-normed ABC mean score is 100 with a standard deviation of 15. We examined 12-month ABC difference scores as well as whether or not the minimal clinically important difference (MCID) in Vineland-II ABC was met. The Vineland-II MCID is estimated to be 2.0–3.75 points, and such, we examined whether or not patients achieved at least a 2.0-point increase in ABC at each 12-month interval [27].

#### Descriptive variables

To characterize the sample, we examined therapeutic service history and family demographic variables. The therapeutic service variables were ABA dose and service history (past receipt of ABA, special education, speech therapy, occupational therapy). The family demographic variables were primary language spoken (recorded into English or Other) and parent marital/partnership status (married or partnered versus single, divorced, or widowed). ABA dose was examined as the percentage of hours or sessions prescribed that were actually received, with a full ABA dose considered to be receipt of at least 80% of prescribed hours or sessions [28]. Demographic variables were patient age (years), gender, and race/ethnicity. Race/ethnicity categories were White, Hispanic, and Other; the ‘other’ category included racial/ethnic groups that had too few participants for individual analysis.

### Analysis

For the analysis of measure concordance, we examined data at 12-month intervals (baseline, 12 months, 24 months) which was the only time interval for which adaptive behavior outcomes were assessed. Descriptive statistics were used to characterize the sample on all outcome variables. We compared differences between dichotomous outcomes (achieving/not achieving clinically meaningful change) at the two study time points (12 months, 24 months) using chi-square tests. To assess concordance among patient progress on goals, caregiver progress on goals, and patient Vineland-II 12-month ABC difference scores, we used a correlation matrix (Pearson *R*) to assess convergent validity and internal consistency reliability.

Our analysis focused on convergent validity because we aimed to assess whether our outcome measures (patient-centered goals, caregiver education and participation goals, adaptive behavior) showed convergent clinically meaningful improvement. We considered the statistical significance and magnitude of correlations within the matrix (*R* < 0.1: small; *R* = 0.1–0.3: medium; *R* > 0.5: large) for all three outcomes [29]. Outcome 1 (percentage of patient-centered goals met) had 4 items; outcome 2 (percentage of caregiver education and participation goals met) had 1 item; and outcome 3 (12-month Vineland-II ABC difference score) had 1 item.

## Results

### Sample description

**Fig. 2:**
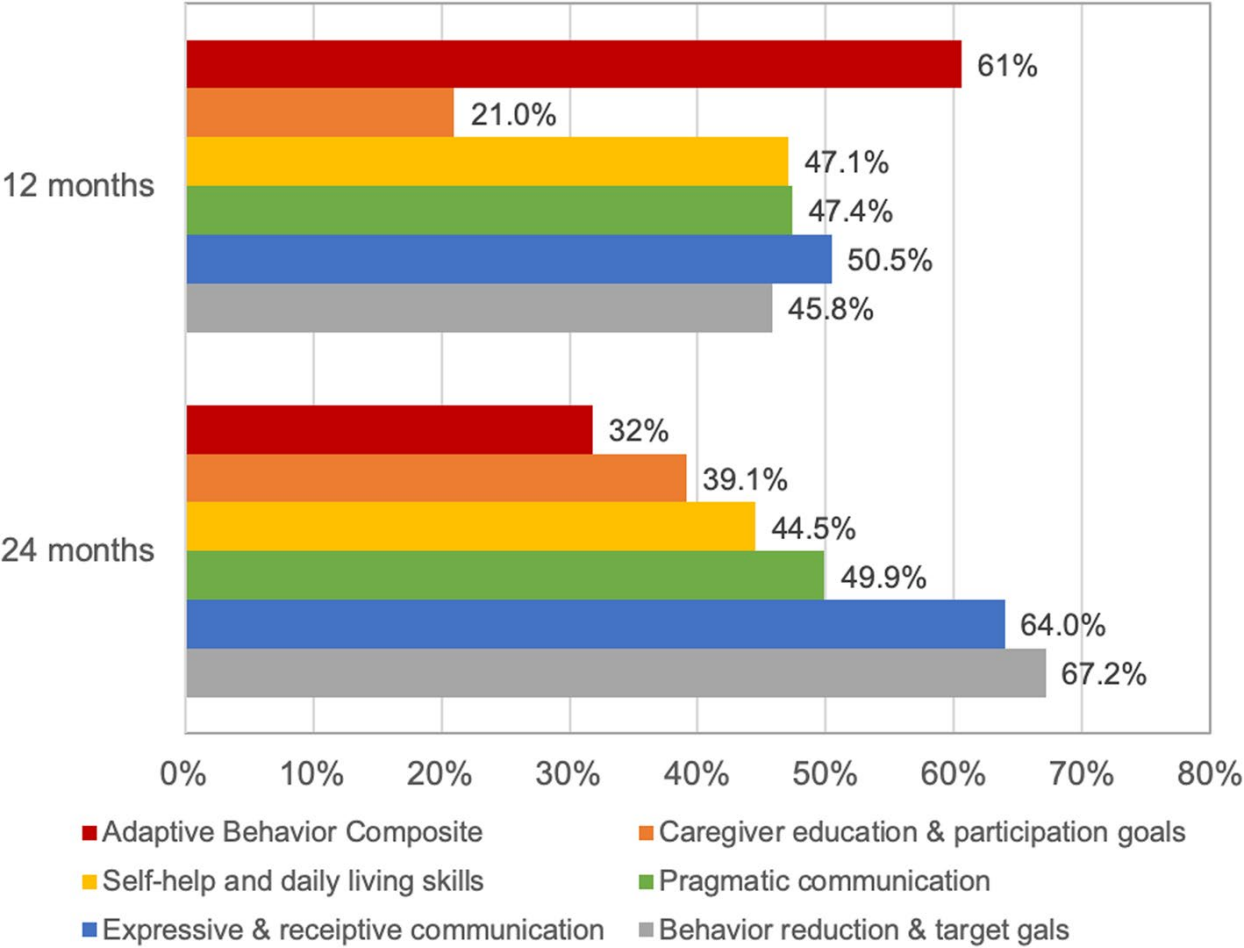
Percentage of Patients Achieving Clinically Meaningful Progress on Outcomes. Legend: This figure shows the percentage of children achieving clinically meaningful progress on patient-centered outcomes and standard adaptive behavior outcomes in a sample of 154 children (3–17 years) receiving Applied Behavior Analysis for Autism Spectrum Disorder over 24 months. The threshold for clinically meaningful progress on adaptive behavior was a 2.0-point or higher increase on the Adaptive Behavior Composite of the Vineland Adaptive Behavior Scales- Second Edition. The threshold for clinically meaningful progress in each of the five individualized patient goal domains (behavior reduction and target goals, expressive and receptive communication, pragmatic communication, self-help and daily living skills, caregiver participation and education) was meeting at least 50% of actively targeted goals, calculated at each timepoint

Characteristics of the analytic subsample with comparison to the original sample are shown in Table 1. The analytic sample was 75.3% boys (*N* = 116) and 50.6% Hispanic (*N* = 78, Table 1), most of whom were commercially insured (65.6%, *N* = 101). The sample was comprised of 16.9% children ages 12–17 years (*N* = 26), 40.4% children ages 7–11 years (*N* = 63), and 42.2% children ages 3–6 years (*N* = 65). Many participants had received services for ASD in the past, including special education (56.5% of the sample, *N* = 87), ABA (22.7%, *N* = 35), occupational therapy (47.4%, *N* = 73), and speech therapy (68.8%, *N* = 106). Full ABA dosing was relatively low, with 27.9% of the sample (*N* = 43) receiving at least 80% of prescribed ABA. There were 136 children (88.3%) who received at least 50% of prescribed ABA. The average number of hours of ABA actually received per week received ranged from 6 to 20 hours (M = 9.5, SD = 7). The percentage of patients who made clinically meaningful adaptive behavior gains decreased between 12 and 24 months of ABA, while the percentage of patients who had meaningful gains on patient-centered goals and parent/caregiver treatment participation goals increased during the same time period (Fig. 2). Comparing differences in achieving clinically meaningful change across outcomes between the 12-month and 24-month time points, three of the six outcomes were statistically significant: ABC difference scores (*P* = 0.04), percentage of pragmatic communication goals met (*P* < .01), and percentage of expressive/receptive communication goals met (*P* < .01).

**Table 1:** Sample description and comparison to original sample

	Original Sample, *N* = 334 N (%)	Analytic Sample, *N* = 154 N (%)
Gender		
Boy	260 (77.8)	116 (75.3)
Girl	74 (22.1)	38 (24.7)
Age group (baseline)		
3 to 6 years	136 (40.7)	65 (42.2)
7 to 11 years	134 (40.1)	63 (40.4)
12 to 17 years	64 (19.2)	26 (16.9)
Race/ethnicity		
White	87 (26.0)	43 (27.9)
Hispanic	161 (48.2)	78 (50.6)
Other	86 (25.7)	33 (21.4)
Insurance		
Commercial	210 (62.8)	101 (65.6)
Medi-Cal	124 (37.1)	53 (34.4)
Primary Language		
English	266 (79.6)	134 (87.0)
Other language	68 (20.4)	20 (13.0)
Parent marital/partnership status		
Married/partnered	209 (62.6)	113 (73.4)
Unmarried/unpartnered	125 (37.4)	41 (26.6)
History of Special Education	219 (65.6)	87 (56.5)
History of ABA	81 (24.3)	35 (22.7)
History of Occupational Therapy	142 (42.5)	73 (47.4)
History of Speech Therapy	201 (60.2)	106 (68.8)
Baseline Adaptive Level		
Low (ABC < 70)	227 (67.6)	91 (59.1)
Moderately low (ABC 70–85)	93 (27.8)	55 (35.7)
Adequate or above (ABC > 85)	14 (4.2)	8 (5.2)

This table shows original sample characteristics for *N* = 334 children ages 3–17 years who received applied behavior analysis (ABA) for Autism Spectrum Disorder in an integrated health system (left column). The analytic sample for this study (*N* = 154) was the subsample of children that received ABA for a full 24 months (right column)

### Correlations and convergent validity among outcome measures

At 12 months, there was low convergent validity between ABC difference score and percentage of patient-centered goals met (Table 2). None of the patient-centered goal domains had statistically significant correlations to ABC difference scores. Internal consistency reliability was poor for patient-centered goals. Two of the four patient-centered goal domains had statistically significant correlations to caregiver education and participation goals (pragmatic communication, *R* = 0.17; self-help and daily living skills, *R* = 0.18); these were medium-magnitude scores. There was no significant correlation between ABC difference score and caregiver education and participation goals met.

**Table 2:** Correlation matrix for patient-centered measures, parent/caregiver measures, and adaptive behavior measures of clinically meaningful progress among children receiving applied behavior analysis

		Behavior reduction and target goals (% met)	Expressive and receptive communication goals (% met)	Pragmatic communication goals (% met)	Self-help and daily living skills (% met)	Caregiver education and participation goals (% met)	Adaptive Behavior Composite (12-month difference score)
	12 months	Outcome 1				Outcome 2	Outcome 3
Outcome 1 (0.50)	Behavior reduction and target goals (% met)	(0.52)	0.16*	0.22*	0.18*	**0.15**	**0.07**
	Expressive and receptive communication goals (% met)		(0.37)	0.46*	0.27*	**0.07**	**0.16**
	Pragmatic communication goals (% met)			(0.37)	0.28*	0.17*	**< 0.01**
	Self-help and daily living skills (% met)				(0.44)	0.18*	**0.07**
Outcome 2 (NA)	Caregiver education and participation goals (% met)					NA	**−0.04**
Outcome 3 (NA)	Adaptive Behavior Composite (12-month difference score)						NA
	24 months	Outcome 1				Outcome 2	Outcome 3
Outcome 1 (0.11)	Behavior reduction and target goals (% met)	(0.38)	−0.14	−0.17*	0.01	**−0.02**	− 0.19*
	Expressive and receptive communication goals (% met)		(−0.05)	0.46	0.12	**0.11**	**0.06**
	Pragmatic communication goals (% met)			(−0.25)	0.12	0.22*	**0.06**
	Self-help and daily living skills (% met)				(0.07)	**0.06**	**−0.14**
Outcome 2 (NA)	Caregiver education and participation goals (% met)					NA	**−0.02**
Outcome 3 (NA)	Adaptive Behavior Composite (12-month difference score)						NA

This table shows a correlation matrix with Pearson *R* correlation coefficients for associations between clinical outcome measures in a sample of 154 children (3–17 years) receiving Applied Behavior Analysis for Autism Spectrum Disorder after 12 months of ABA and after 24 months of ABA (*Value is significant at the 0.05 level). Outcome 1 is clinical improvement on patient-centered goals; Outcome 2 is clinical improvement in caregiver/parent goals; and Outcome 3 is clinical improvement in adaptive behavior. Bold values are those where convergent validity is expected (homotrait-heteromethod). Parentheses indicate internal consistency reliability (Cronbach’s alpha) for the outcome if the item where the parentheses are located was excluded (for outcomes with only 1 item, no internal consistency reliability was calculated). The overall internal consistency reliability for each outcome is shown in the table subheadings

At 24 months, convergent validity was also low. One patient-centered goal domain had a medium-magnitude and significant negative correlation to ABC difference score (behavior reduction and target goals, *R* = -0.19). Internal consistency reliability was poor for patient-centered goals. One patient-centered goal domain was significantly correlated with caregiver education and participation goals (pragmatic communication, *R* = 0.22). No correlation was observed between ABC difference score and caregiver education and participation goals at 24 months.

## Discussion

In this investigation of concordance between patient-centered measures of clinical progress in a single health system, caregiver education and participation in treatment goals, and a standard measure of adaptive behavior (i.e., Vineland-II ABC 12-month difference score) used in routine practice for children receiving ABA, we found limited concordance among measures. Our data showed medium concordance in two of nine measures at 12 months and medium concordance in two of nine measures at 24 months. The percentage of children achieving clinically meaningful gain on patient-centered goal measures increased between 12 and 24 months of ABA, while the percentage of children achieving clinically meaningful gains in adaptive behavior declined during the same time period. Limited convergent validity was found among the measures at both 12 and 24 months. This investigation used a clinical sample rather than a research sample, and as such, our findings point to the challenges of measuring and improving meaningful patient outcomes when ABA is delivered in real-world settings.

Our findings suggest that measure concordance may be limited during real-world implementation of ABA, due to the confluence of limited research on measurement of patient goals, sub-optimal ABA dosing, and patient heterogeneity. In other community and health system implementations of ABA, sub-optimal dosing is common due to barriers such as time demands on families, difficulty affording co-pays, or preferences for other services [22, 30]. It is important for researchers and clinicians to this context (i.e., that full dosing may be unlikely in real-world settings) when selecting measures of treatment progress. Current clinical practice guidelines for ABA place a strong emphasis on setting and tracking individualized, measurable patient goals with input from parents, caregivers, patients, or other stakeholders [8]. These goals can have implications for treatment continuation and funding. At the same time, standard measures like the Vineland-II may also be required for insurance reimbursement. The divergence between patient-centered and standard measures found in this study suggests a need to reconsider how to best measure treatment progress in in real-world healthcare and community settings, from the perspective of both clinicians and payers. The Vineland-II has limitations for detecting small treatment gains or capturing patient-centered outcomes, and as such, there might be missed opportunities to meet patient and family needs if relying on the Vineland-II alone.

There are several other possible explanations for the lack of concordance among patient-centered outcome measures and a standard adaptive behavior measure. Although we theoretically expected to see convergent gains on several indicators of clinically meaningful progress, it is possible that progress on individualized patient goals may follow a different trajectory than gains in the Vineland-II ABC. This may be especially true for ABA prescribed for medically necessary skill development. Prior studies have suggested that ABA results in positive changes across multiple outcome domains, but that the magnitude of gains is variable across outcome domains and by ABA intensity and duration [30, 31]. Outcome gains may also vary by age, with decelerating gains as children age [18, 32]. The rate of clinically meaningful adaptive behavior gains observed early in ABA may begin to decelerate by 24 months for these reasons, while patient-centered goals that were more flexible and frequently modified based on individual patient progress (in the health system used in our study, every 6 months) could continue to show growth. Similarly, clinicians may gain familiarity with children over time and set goals that are more achievable, resulting in more goals mastered during the 12–24 month than the 0–12 month period. Another explanation for the discordance we observed is that patient-centered goals may become more important over time in treatment as families adapt expectations of ABA and what goals are realistic for their child to meet [33]. Over time in ABA, parents may shift towards a focus on goals that are more achievable and meaningful for their individual child.

Our findings may also be influenced by the reporters of patient-centered outcomes data in this health system. Parents/caregivers reported independently on child adaptive behavior, while patient-centered goals were set and reported on by both parents/caregivers and BCBAs®. As such, the Vineland-II ABC may have been biased towards under-estimation of patient progress out of parent desire for service continuation, while the patient goals measure reported by BCBAs® may have been biased towards over-estimation out of desire for documenting treatment efficacy and clinical improvement [34]. Parents of children with ASD hold a wide range of beliefs about the cause of ASD and the benefits of various treatment modalities, and they also have different levels of engagement in ABA treatment [35]. These beliefs—as well as past experiences with ABA and other treatment modalities—may influence how parents perceive their child’s progress or level of improvement in ABA. Research on concordance between parents/caregivers and BCBAs in measuring patient-centered outcomes of ABA is limited, and more studies are needed to understand differences in perceptions of clinically meaningful progress. Future research should include additional validated measures of constructs that are meaningful to patients and families (e.g., quality of life), as well as whether age and developmental stage affect measure sensitivity to change.

Overall, our findings around limited concordance suggest that more research is needed to understand how to optimally measure and improve patient-centered outcomes. However, there are some clinical considerations arising from our results. To improve concordance in clinical care and track patient progress in multiple domains, clinicians should first identify patient and family treatment goals and priorities; and then select corresponding measures that are sensitive to change. Realistically, reaching consensus on how to define clinically meaningful progress or which domains of progress are valuable to track may be challenging and there may be disagreement among stakeholders (e.g., payers, health system leaders, clinicians, parents). A truly patient-centered measurement approach would require structural support from health systems (e.g., clinical guidance on validated treatment measure options and use; integration of measures in electronic health records; dashboard for tracking outcomes over time) and health system prioritization of patient-centered care.

This investigation has strengths and limitations that should be considered in interpreting the findings. We used a diverse sample and multiple measures of patient progress on clinically meaningful outcomes. We compared a gold-standard measure of adaptive behavior, the Vineland-II, to patient-centered goals measures to explore concordance. Although the Vineland-II is widely used as an outcome measure in clinical practice, it was not originally designed to be an outcome measure and has been shown to have low sensitivity for detecting treatment gains in some prior studies [36]. It is possible that a more sensitive measure or measurement of other domains of function (e.g., challenging behavior, maladaptive behavior, sub-domains of adaptive behavior) may have shown better concordance with goals mastered, but such measures were not available in clinical data used in this study. We selected a random, representative sample of patients with ASD in Southern California so that our findings around concordance can be generalized to a non-research population of children with ASD. Limitations of this investigation are that the sample was a clinical sample of children who may have had more severe ASD than the general ASD population. We did not capture outcomes for patients who discontinued ABA, and we did not have measures of maladaptive behavior or detailed records on school services. Finally, the MCIDs used in this study are determined at an aggregate level thus may not capture individual variation in what families consider to be meaningful treatment change.

## Conclusion

When ABA is widely implemented in a health system, there is limited concordance between patient-centered goals measures, caregiver treatment participation goals measures, and the Vineland-II ABC on clinically meaningful treatment progress for children with ASD. Although concordance among various patient outcome measures has been shown in controlled research studies, finding agreement about what constitutes clinically meaningful progress is much more challenging in real-world health system implementation of ABA. Thus, it is important to use multiple measures and informants of patient progress that can be documented, compared, and used to inform treatment decisions in integrated health information systems. Clinicians should have ongoing dialogue with patients, parents/caregivers, and families to ensure that interventions like ABA are resulting in progress towards outcomes that are meaningful to patients and families. This dialogue is essential to ensure that individualized patient goals—which are heavily emphasized by practice guidelines, health systems, and payers—are actually measuring something meaningful in the lives patients.

## Data Availability

The datasets analyzed during the current study are not publicly available because they include protected medical records data. Data inquires should be directed to the corresponding author.

## Abbreviations

ABA: Applied behavior analysis
ABC: Adaptive behavior composite
ASD: Autism spectrum disorder
BCBA®: Board Certified Behavior Analyst®
